# MATWIN: bridging the gap between academic research and industry

**DOI:** 10.1186/s40246-015-0045-z

**Published:** 2015-09-16

**Authors:** Josy Reiffers, Lucia Robert

**Affiliations:** MATWIN, Institut Bergonié, 229 cours de l’Argonne, CS 61283, 33076 Bordeaux cedex, France

## Abstract

MATWIN (Maturation and Accelerating Translation With INdustry) is part of the nationwide effort to support cancer innovation. This unique program is willing to support innovative research projects providing tools, resources, and staff dedicated to project leaders wishing to optimize the industrial attractiveness of their project. The overall objective is clear: fight cancer always more effectively.

## Origins

**Fig. 1:**
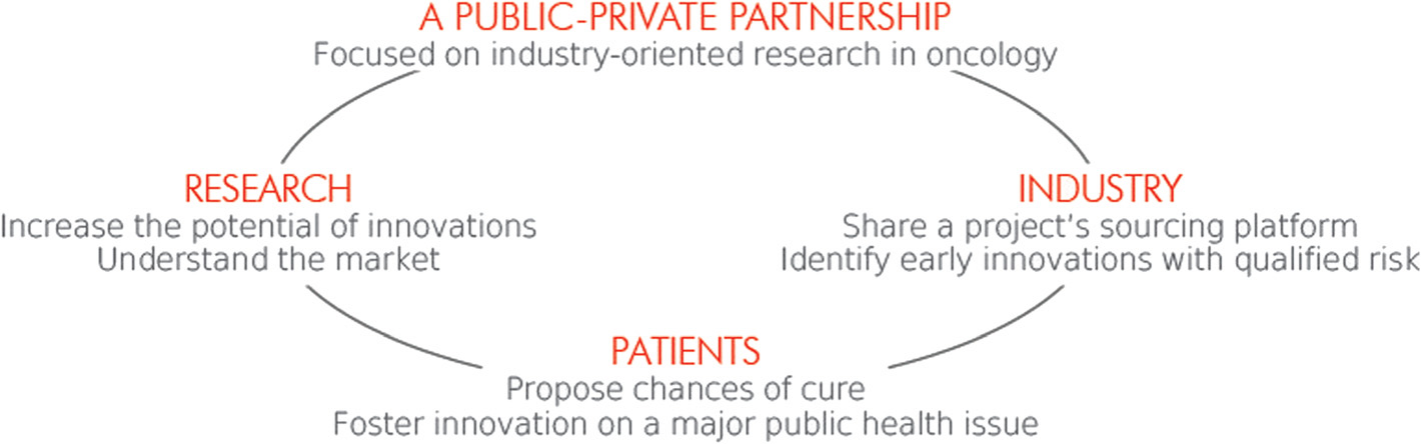
MATWIN: linking academia, industry, and patients

The MATWIN platform, *M*aturation and *A*ccelerating *T*ranslation *W*ith *In*dustry, was created in 2009 in Bordeaux (France), ensuing discussions at the Greater South West Cancéropôle, one of the seven French Cancéropôles, who aim to enable better coordination of skills and resources in cancer research at both the regional and inter-regional levels. MATWIN was founded to facilitate collaboration between academics and industry, which are typically two very separate worlds with different concerns, especially in France. MATWIN’s goal is to bridge the gap between the Cancer research community and the pharmaceutical industry, thus aiming to accelerate the innovation process from bench to the bedside (Fig. 1).

Cancer is the leading cause of death in France [1]. One thousand new cancer diagnoses are made daily in France, and around 400 people per day die from their cancer. Cancer incidence continues to rise, yet, cancer mortality has decreased over the last 20 years, thanks to advances in therapeutics and diagnostics. Much is at stake which is the reason why France has set up three successive Cancer Plans since 2003. The third one was launched in 2014 and will make it possible to reinforce advances, open new paths, and engage innovative project initiatives, such as MATWIN.

### MATWIN process

The MATWIN platform is based on a public-private partnership bringing together the seven French Cancéropôles and 11 of the biggest pharmaceutical companies worldwide: Amgen, AstraZeneca, Bayer Healthcare, Bristol-Myers Squibb, Celgene, GSK, Merck Serono, Novartis, Pierre Fabre, Roche, and Sanofi. This nationwide program is exclusively dedicated to the maturation of early oncology research projects up to the preclinical proof of concept. Since January 2011, the program has been managed through a private society MATWIN SAS, with offices located in Bordeaux, which is now a wholly-owned subsidiary of the UNICANCER group gathering the 20 French Comprehensive Cancer Centers. Using Cancéropôles’ networks, MATWIN experts identify and qualify patented French academic research projects in oncology to reinforce proof of concept and preclinical maturation before transfer to the pharmaceutical industry. This kind of initiative may be familiar to American researchers and industries, but it is quite innovative and unique in continental Europe.

**Fig. 2:**
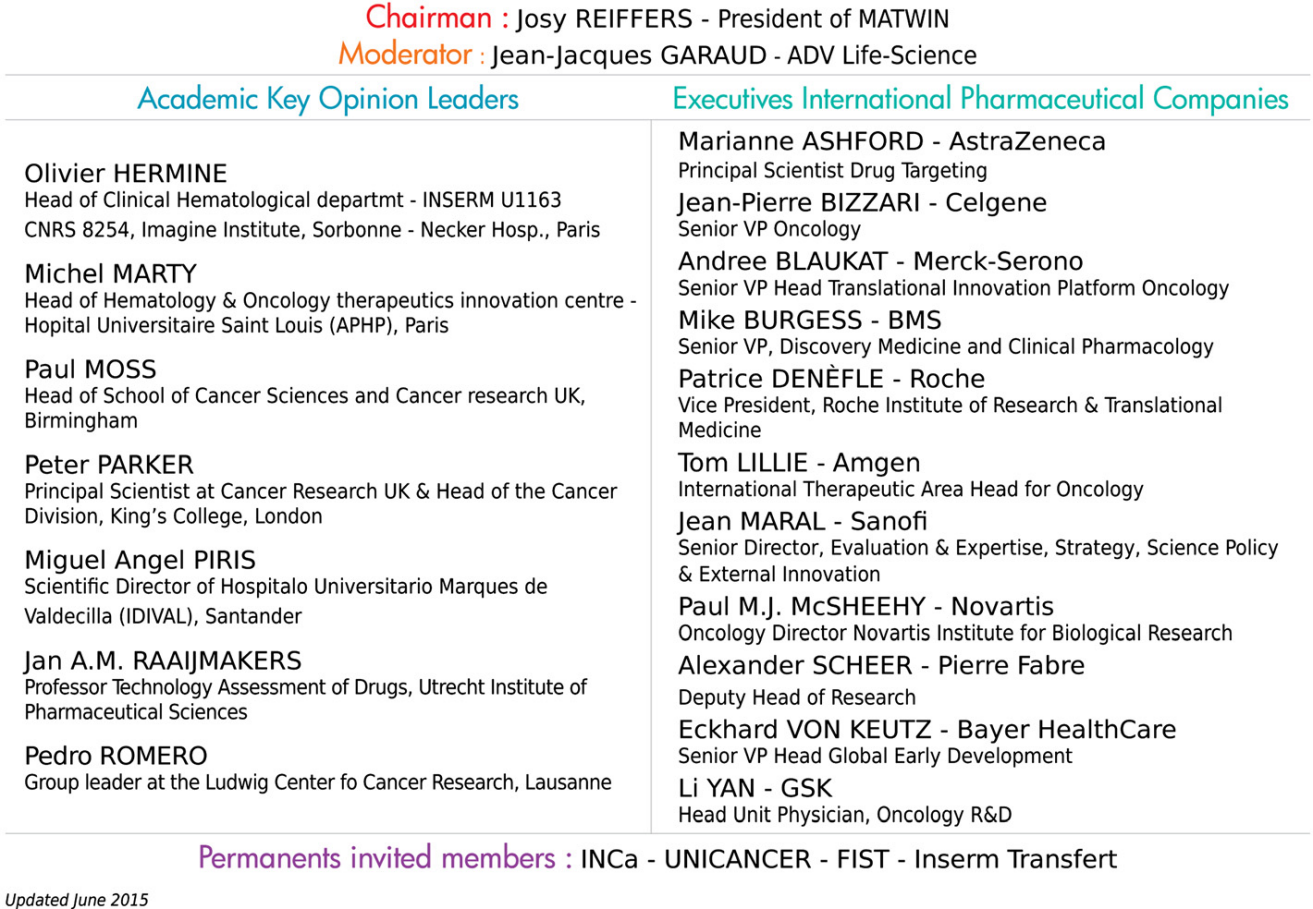
MATWIN Board composition

The annual process starts with a nationwide call for proposals, with a selection of projects based on strict eligibility criteria. Eligible projects are then submitted for both academic and industrial assessment. Individual working sessions are organized to increase industrial attractiveness of projects. The selection process ends in Bordeaux, where individual project auditions are held in Bordeaux with an international public-private board who recommends projects matching specific criteria to receive the “MATWIN label” (Fig. 2).

The MATWIN label acts like a stamp of approval for projects, making them more attractive for collaborations or partnerships with MATWIN industrial partners or external investors. During the whole process, academic teams are encouraged to be aware of economic challenges and to better understand the market expectations.

## Results

The process has already proved to be successful: 6 years since the creation of the MATWIN platform, more than 50 % of the projects selected for final audition by the MATWIN Board have led to first discussions with industrial partners (MATWIN partners or not) or have been restructured into start-ups. Of course, creating a company is a way of simplifying the discussion with industrial partners, with clearer issues of trusteeship, and this is also a mark of a research leader’s credibility and commitment to the valorization of the research project.

During the 2009–2015 period, MATWIN has led to the following achievements:

- 98 letters of intent received
- 57 project applications
- >200 academic and industrial assessments
- 33 projects submitted to the MATWIN Board
- 16 projects have led to first industrial interest
- >15 start-ups created
- three projects in ongoing maturation process with industrial partners
- and many other discussions in progress.

### A win-win partnership

Through MATWIN, all program stakeholders are committed to a win-win partnership:

- By integrating the MATWIN program, the project leader benefits from project assessment (three to five international industrial and academic assessments per project), individual high value-added coaching with pharmaceutical development specialists, and the opportunity to present the project to a board that is unique in Europe with top executives from pharmaceutical companies and oncology key opinion leaders. The MATWIN process also offers a significant pedagogic added-value opportunity to appropriate tech-transfer issues.
- By accompanying the project leader during the whole MATWIN process, technology transfer offices have an excellent opportunity to strengthen and legitimize their possible funding decision on the project.
- For industrial partners, MATWIN represents a privileged access to an early innovation shared platform.

MATWIN thus acts as a clear single window process in cancer research that contributes to accelerate innovation transfer to the benefit of patients on a major public health issue. It has long been said that France lagged behind countries like the USA regarding research public-private partnerships which have been part of the economic landscape and research culture for a long time in the USA. France is now clearly trying to make up for this delay, which is one more reason to encourage an innovative program such as MATWIN to bridge once and for all the gap between academic research and industry.

If you need further information, please visit our website: http://www.matwin.org.

## References

[1] INCa. les Cancers en France en 2014. Paris, INCa. 2014.

